# Decisions among the Undecided: Implicit Attitudes Predict Future Voting Behavior of Undecided Voters

**DOI:** 10.1371/journal.pone.0085680

**Published:** 2014-01-29

**Authors:** Kristjen B. Lundberg, B. Keith Payne

**Affiliations:** Department of Psychology, University of North Carolina at Chapel Hill, Chapel Hill, North Carolina, United States of America; University of Queensland, Australia

## Abstract

Implicit attitudes have been suggested as a key to unlock the hidden preferences of undecided voters. Past research, however, offered mixed support for this hypothesis. The present research used a large nationally representative sample and a longitudinal design to examine the predictive utility of implicit and explicit attitude measures in the 2008 U.S. presidential election. In our analyses, explicit attitudes toward candidates predicted voting better for decided than undecided voters, but implicit candidate attitudes were predictive of voting for both decided and undecided voters. Extending our examination to implicit and explicit racial attitudes, we found the same pattern. Taken together, these results provide convergent evidence that implicit attitudes predict voting about as well for undecided as for decided voters. We also assessed a novel explanation for these effects by evaluating whether implicit attitudes may predict the choices of undecided voters, in part, because they are neglected when people introspect about their confidence. Consistent with this idea, we found that the extremity of explicit but not implicit attitudes was associated with greater confidence. These analyses shed new light on the utility of implicit measures in predicting future behavior among individuals who feel undecided. Considering the prior studies together with this new evidence, the data seem to be consistent that implicit attitudes may be successful in predicting the behavior of undecided voters.

## Introduction

What does it mean when someone says they are undecided about how to vote just weeks before an important election? Do undecided voters truly have no clear preferences? Do they have preferences, but are still weighing all the information at hand? Or, do they simply lack conscious insight into what their preferences are? Regardless, undecided voters are hard to ignore. Less than two months before the 2012 U.S. presidential election, five percent of voters in swing states were undecided, and another 17% said they might change their minds [1]. These percentages are more than enough to decide most elections, making the prediction of undecided voters' behavior an important problem for pollsters and social scientists. Is it possible to predict the behavior of individuals who cannot predict their own?

Psychologists have recently suggested an innovative solution to this problem. Galdi, Arcuri, and Gawronski [2] proposed that a key to predicting the behavior of undecided voters lies in the distinction between explicit and implicit attitudes. Explicit attitudes are evaluations of topics that are consciously endorsed and voluntarily reported. Traditional self-report surveys, including the questions asked in polls, measure explicit attitudes. In contrast, implicit attitudes are spontaneous evaluations that are automatically evoked when encountering or contemplating an issue. Implicit evaluations come to mind whether or not they are endorsed as true. They may affect behavior even when their influence is unwanted.

Galdi and colleagues [2] proposed that explicit attitudes are stronger predictors of behavior for decided voters than undecided voters, but implicit attitudes are stronger predictors for undecided voters than decided voters. They reasoned that implicit attitudes may indirectly influence future voting behavior by biasing the processing of decision-relevant information. To the extent that people use this biased set of information in making a deliberate decision, implicit attitudes may predict eventual decisions even though respondents are undecided when the implicit attitudes are measured. Evidence for this hypothesis was reported by Galdi, Gawronski, Arcuri, and Friese [3], who demonstrated that the implicit attitudes of undecided participants predicted selective exposure to information consistent with their implicit attitudes. Individuals who describe themselves as undecided may therefore have implicit attitudes that will ultimately lead to conscious preferences, but have not yet done so (see also [4]).

There is additional reason to believe that implicit attitudes may predict behavior for undecided voters: Being “decided” or “undecided” is a metacognitive judgment that people make about their own decision processes. Models of explicit and implicit attitudes suggest that people are more likely to consider their explicit attitudes to be a valid basis for judgment [5], [6]. Whereas explicit attitudes are experienced as conscious preferences or “considered opinions,” implicit attitudes tend to be experienced as “gut feelings” [5], [7]. Further, those who consider their automatic associations to be a less valid source of information have been shown to exhibit weaker correspondences between explicit and implicit measures [8], [9], [10], [11].

We suggest that, when people indicate whether they have decided, they are more likely to base this judgment on their consciously endorsed (i.e., explicit) attitudes than on implicit attitudes. If explicit attitudes strongly favor one option over the other, then the respondent will claim to be decided, whereas if the explicit attitude is weak, unclear, or ambivalent, then the respondent will claim to be undecided. Based on this reasoning, explicit attitudes should be more predictive of behavior among decided voters than undecided voters. Introspection, however, may overlook implicit attitudes, especially if those implicit attitudes are considered a less trustworthy source of information by default. If so, then when people introspect about whether they have decided, they may focus on consciously endorsed attitudes and neglect implicit attitudes. Implicit attitudes would nonetheless be automatically activated and would still have the potential to influence behavior. Based on this introspective neglect hypothesis, implicit attitudes should be equally predictive of behavior among the decided and the undecided.

The introspective neglect account and the biased processing account make different predictions about whether implicit attitudes should predict behavior for decided voters after explicit attitudes have been accounted for. Under the biased processing account, implicit attitudes lead to deliberate decisions only indirectly, by biasing the formation of the deliberate decision. Once the biased information search has led to a deliberate decision, no direct effect of implicit attitudes is predicted over and above the effects of explicit attitudes. Both accounts thus predict that implicit attitudes should predict decisions among the undecided, but only the introspective neglect account predicts that implicit attitudes should be similarly predictive among decided voters after removing the effects of explicit attitudes.

### Do Implicit Attitudes Predict Future Behavior of Undecided Voters?

The theoretical reasons that implicit attitudes might predict the behavior of undecided voters are clear, but the empirical evidence to date has been mixed. In this section, we review the results of the most relevant previous studies. One study by Arcuri and colleagues [12] examined implicit political attitudes in the 2001 Italian general election. This study found that implicit attitudes predicted voting intentions for both decided and undecided voters, consistent with the idea that voters neglect implicit attitudes when they assess whether they are decided. Based on this finding, implicit attitude measures should be useful in predicting the behavior of undecided voters even if the voters themselves do not yet know how they will vote. However, this study did not simultaneously account for explicit measures and, therefore, cannot speak to the unique predictive power of implicit measures.

A second study by Galdi and colleagues [2] compared implicit and explicit attitudes toward a politically charged issue in an Italian sample. In separate analyses for decided and undecided participants, this study found that explicit, but not implicit, attitudes were predictive of future choices among decided participants. In contrast, implicit, but not explicit, attitudes were predictive of future choices among undecided participants. This pattern suggests that voters rely on explicit attitudes when assessing whether they have decided, but when they are undecided they rely more on implicit attitudes. These analyses, too, suggest that implicit attitude measures should be especially useful in predicting the behavior of undecided voters. However, in multiple regression analyses that treated decidedness as a dummy-coded moderator variable, explicit attitudes were not moderated by confidence in predicting future choices, while implicit attitudes were. While this finding corroborates the account that implicit attitudes may be more predictive for undecided than decided voters, it also suggests that explicit attitudes may be equally predictive for both decided and undecided voters.

Finally, Friese and colleagues [13] examined the ability of implicit and explicit attitude measures to predict voting behavior in the 2008 presidential election in the United States and the 2009 parliamentary election in Germany. Across three sets of multiple regression analyses that treated decidedness as a moderator variable and examined implicit attitude measures separately, implicit attitudes were consistently predictive of voting behavior, and they were more predictive for decided than undecided respondents (in contrast to Galdi et al.'s [2] findings). However, the variance due to explicit attitudes and their interaction with decidedness was not removed in these analyses. Because explicit and implicit attitudes were strongly correlated in these samples (*r*s between .52 and .72), implicit attitudes may have spuriously appeared to be more predictive among decided than undecided voters because of the shared variance with explicit attitudes.

Indeed, once explicit attitudes were added as predictors to the models, a different pattern emerged: In Study 1 (as reported in their Table 3), explicit attitudes toward U.S. presidential candidates were more predictive for decided than undecided voters, but implicit attitudes were no longer a significant predictor of the vote. In Study 2 (as reported in their Table 7), explicit attitudes toward German parliamentary candidates were more predictive for decided than undecided voters, while implicit attitudes were predictive of voting for both decided and undecided voters. Finally, a second analysis in Study 2 (as reported in their Table 5) examined explicit and implicit attitudes toward political camps rather than candidates. In this case, both explicit and implicit attitudes were predictive of voting for both decided and undecided voters.

Taken together, two out of the three critical analyses in Friese et al. [13] suggest that explicit attitudes may be more predictive for decided than undecided voters. Implicit attitudes were equally predictive for decided and undecided voters in all three analyses, but the effects of implicit attitudes were small in all analyses and non-significant in one. It was these small effects of implicit attitudes that led Friese and colleagues to question the utility of implicit measures in forecasting behavior among the undecided.

### The Present Research

In this study we re-examine the roles of implicit and explicit attitudes in predicting voting behavior among decided and undecided voters. Like Friese and colleagues [13], we studied actual voting behavior in a major national election (the 2008 U.S. presidential election). Whereas previous studies used convenience samples or opt-in samples, ours is the first to address these questions using a large nationally representative sample. We began our re-examination of the findings of previous studies [2], [12], [13] by modeling the relationships between implicit and explicit attitudes toward the presidential candidates, decidedness, and voting behavior (Analysis 1).

Our study provided a second opportunity to test the relationships of interest by examining implicit and explicit racial attitudes (Analysis 2). The nomination of Barack Obama in 2008 offered an unprecedented occasion to study the role of implicit and explicit racial attitudes in voting for a Black presidential candidate. We replicated the analyses of candidate attitudes using these racial attitude measures and explored for the first time whether implicit and explicit racial prejudice predicted voting differently for decided versus undecided voters.

Finally, we tested a hypothesis implied by the introspective neglect account (Analysis 3). If metacognitions about whether one has decided are based on explicit but not implicit attitudes, then more extreme explicit attitudes should be associated with greater confidence. This explanation predicts a curvilinear relationship between explicit attitudes and confidence, with more extreme attitudes in favor of either voting option associated with high confidence. Such a curvilinear relationship between confidence and implicit attitudes is expected to be smaller or absent if implicit attitudes are neglected during introspection.

Our study used different measures of implicit attitudes and decidedness than previous studies. Whereas previous studies [2], [12], [13] measured implicit attitudes using the Implicit Association Test (IAT) [14], our study uses the Affect Misattribution Procedure (AMP) [15]. Although the IAT and the AMP are both well validated implicit measures, past research has generally found the two measures to be only weakly correlated [16], [17]. This may be, first, because the AMP measures affective responses to the stimuli presented as primes whereas the IAT is more likely to measure associations to the category labels. Second, the mechanism driving the AMP is assumed to be a misattribution of affect from the prime to the target, whereas the mechanism driving the IAT is assumed to be response interference. A third difference is that the measure of interest in the AMP is an evaluation, whereas in the IAT it is response times. Thus, the two measures are likely to differ for both psychological and technical reasons.

Additionally, the previous studies we reviewed measured decidedness with a binary measure in which respondents classified themselves as decided or undecided [2], [12], [13]. Our study utilized a continuous measure of confidence in one's vote. The two measures differ in that our item did not allow subjects to make the determination of whether they considered themselves to have decided. With a binary measure, two respondents with identical levels of confidence may classify themselves differently if they apply different thresholds. The continuous measure of confidence avoids the threshold problem and may, therefore, provide a more precise measure of how strongly participants feel about their decision.

The difference in measures across studies suggests that our results may not be directly comparable to existing findings, and the results should not be interpreted as a direct replication attempt. Nonetheless, the underlying psychological questions that we are addressing are the same. These new data are well positioned to provide additional evidence on the important question of whether (and why) implicit attitudes may be effective predictors of decisions among respondents who are as yet undecided.

In summary, the present study tested (1) whether implicit attitudes toward Barack Obama and John McCain predicted voting among undecided voters, (2) whether implicit racial attitudes predicted voting among undecided voters, and (3) whether confidence in voting decisions was more strongly associated with the strength of explicit than implicit attitudes. Together, these data shed new light on the utility of implicit measures in predicting decisions among the undecided.

#### Respondents and sampling

In all analyses described, we used data from the American National Election Studies (ANES) 2008–2009 Panel Study. For this study, panel respondents were recruited by telephone using random digit dialing to participate in an Internet-based study. Individuals who lacked a computer or Internet access at home were provided them at no cost. Respondents were compensated for completing monthly Internet surveys from January 2008 through August 2009. All analyses utilized sampling weights in order to correct for unequal probabilities of selection and nonresponse bias, as well as the Taylor Series method of calculating sampling errors and conducting significance testing in order to account for the clustering of the sample. These design-consistent estimation procedures allow us to generalize our findings to the American electorate. For additional information on the panel study, its sampling and recruitment techniques, and its procedures for the calculation of weights, please see DeBell, Krosnick, and Lupia [18].

#### Ethics statement

Data collection for the ANES 2008–2009 Panel Study was performed by Knowledge Networks, Inc., under a contract with Stanford University and with approval from the Stanford University Institutional Review Board (IRB). Knowledge Networks conforms to the Code of Standards and Ethics for Survey Research of the Council of American Survey Research Organizations, and all participants provided informed consent prior to participation. Additionally, the University of North Carolina at Chapel Hill Non-Biomedical IRB determined this research to be exempt from review for human subjects research (#08-0805).

## Analysis 1

In this first analysis, we began our re-examination of the role of implicit attitudes among undecided voters by predicting voting behavior from implicit and explicit attitudes toward the presidential candidates.

### Measures

#### Implicit candidate preference

Implicit candidate preference was measured using the Affect Misattribution Procedure (AMP) [15]. The measure was administered in either September or October 2008 (date of completion was determined randomly for each respondent). Participants completed 48 trials, each of which began by presenting a fixation cue, followed by a photograph of either Barack Obama or John McCain presented for 75 ms, followed by the appearance of a randomly assigned Chinese ideograph for 250 ms. Finally, the ideograph was replaced by a visual mask composed of black and white dots in a random “noise” pattern. The mask remained on the screen until a response was made. Respondents were instructed to judge whether each ideograph was pleasant or unpleasant while avoiding influence from the photographs. Unintentional influence of the primes on judgments can be used to measure attitudes toward the candidates pictured. Previous research has shown that the procedure is a reliable and valid measure of implicit attitudes [15], [19], [20]. Implicit candidate preference was calculated by subtracting the proportion of pleasant judgments that followed photographs of Mr. McCain from the proportion of pleasant judgments that followed photographs of Mr. Obama (*α* = .95). Higher scores indicate a greater implicit preference for Mr. Obama.

#### Explicit candidate preference

Explicit candidate preference was measured using two items assessing liking for Mr. Obama and Mr. McCain. Respondents were asked to indicate the extent to which they liked each candidate in a branching question (“Do you like John McCain [Barack Obama], dislike him, or neither like nor dislike him?”; then “Do you (dis)like him a great deal, a moderate amount, or a little?”). Liking for Mr. McCain was subtracted from liking for Mr. Obama, such that higher scores indicate a greater explicit preference for Mr. Obama. Explicit attitude measures were collected during the same survey wave as the implicit attitude measure.

#### Confidence regarding one's voting intention

To assess whether respondents had decided about their vote, respondents were first asked for whom they thought they would vote in the election for president. After answering, they were then asked: “How sure are you of that?” Responses were made on a 5-point scale from “extremely sure” to “not sure at all.” We scored the item such that higher scores indicate greater confidence in one's voting intention. Confidence was measured in the same wave as that in which the attitude measures were collected.

#### Voting behavior

Respondents were surveyed in November after the election and were asked to report whether they voted for president and, if so, for whom they had voted. For our analyses, responses were scored such that 0 indicated a vote for Mr. McCain and 1 indicated a vote for Mr. Obama. All other responses (i.e., those who did not vote for president or voted for a third-party candidate) were not included in our analyses.

### Results

Of those respondents who completed all measures of interest (*N* = 2,013), 52.5% indicated a vote for Mr. Obama (47.5% for Mr. McCain). Additionally, 60.2% indicated that they were “extremely sure,” 18.2% that they were “very sure,” 14.4% that they were “moderately sure,” 4.1% that they were “slightly sure,” and 3.1% that they were “not sure at all.”

For all analyses, continuous variables were standardized using *z*-scores prior to analysis. Additionally, because respondents were completing the predictors of interest in both September and October 2008, a dummy variable indicating date of administration was included in all analyses, coded as 0 (September 2008) and 1 (October 2008), but coefficients are reported only for the attitudes and confidence variables that are of interest for the hypotheses tested.

Model 1 in Table 1 features the results of a logistic regression analysis predicting votes for Mr. Obama (versus Mr. McCain) from explicit candidate attitudes and their interaction with confidence. As expected, as explicit preference for Mr. Obama increased, respondents were significantly more likely to vote for Mr. Obama (*B* = 4.530, *SE* = .500, *p*<.001). The interaction between confidence and explicit candidate preference also was significant (*B* = 1.343, *SE* = .326, *p*<.001), indicating that explicit attitudes were more predictive of voting behavior for those who were more confident about how they would vote.

**Table 1:** Results of logistic regression analyses predicting voting behavior from explicit and implicit candidate preference and confidence.

	Model 1: Explicit Candidate Attitudes					Model 2: Implicit Candidate Attitudes					Model 3: Explicit and Implicit Candidate Attitudes				
	% CCC = 87.9					% CCC = 78.1					% CCC = 90.3				
Variable	*B*	*SE*	Wald	*p*	OR	*B*	*SE*	Wald	*p*	OR	*B*	*SE*	Wald	*p*	OR
Constant	0.258	0.195	1.749	0.186	1.294	0.247	0.114	4.693	0.303	1.280	0.382	0.220	3.011	0.083	1.466
Explicit Attitudes	4.530	0.500	82.202	<.001	92.731						3.718	0.473	61.880	<.001	41.197
Implicit Attitudes						3.011	0.330	83.109	<.001	20.302	2.179	0.351	38.635	<.001	8.834
Confidence	−0.046	0.098	0.218	0.641	0.955	−0.022	0.077	0.083	0.773	0.978	0.005	0.107	0.003	0.960	1.005
Explicit*Confidence	1.343	0.326	16.997	<.001	3.830						1.215	0.324	14.050	<.001	3.371
Implicit*Confidence						0.608	0.244	6.216	0.013	1.837	0.362	0.239	2.288	0.130	1.436

Predicting votes for Mr. Obama (1) versus Mr. McCain (0) from explicit and implicit preference for Mr. Obama (versus Mr. McCain) and their interaction with confidence. Controlling for date of attitude measures administration. Model 1 examines explicit candidate attitudes separately (*N* = 2,058). Model 2 examines implicit candidate attitudes separately (*N* = 2,013). Model 3 examines both attitude measures simultaneously (*N* = 2,013). CCC: correctly classified cases; *B*: regression weight *B* (log odds); *SE*: standard error of the regression weight *B*; Wald: Wald test statistic; OR: Odds ratio. Relative amount by which the odds increase (OR >1.0) or decrease (OR <1.0) when the value of the predictor is increased by 1 SD.

The second model in Table 1 shows a parallel logistic regression analysis examining implicit attitudes. Respondents with greater implicit preference for Mr. Obama were significantly more likely to vote for him (*B* = 3.011, *SE* = .330, *p*<.001). Further, the interaction between confidence and implicit candidate preference was significant (*B* = .608, *SE* = .244, *p* = .013), and the effect was in the same direction as for explicit attitudes. These results are consistent with Friese et al.'s [13] finding that, when modeled separately, both explicit and implicit attitudes were more predictive of voting behavior for decided than for undecided voters.

The results thus far examined explicit and implicit attitudes separately. However, explicit and implicit candidate preferences were highly correlated (*r* = .688, *p*<.001). As noted previously, this shared variance between explicit and implicit attitudes makes it important to investigate the unique effects of each measure when the other is statistically controlled. The third model in Table 1 shows that explicit (*B* = 3.718, *SE* = .473, *p*<.001) and implicit (*B* = 2.179, *SE* = .351, *p*<.001) candidate preferences each uniquely predicted voting.

**Figure 1:**
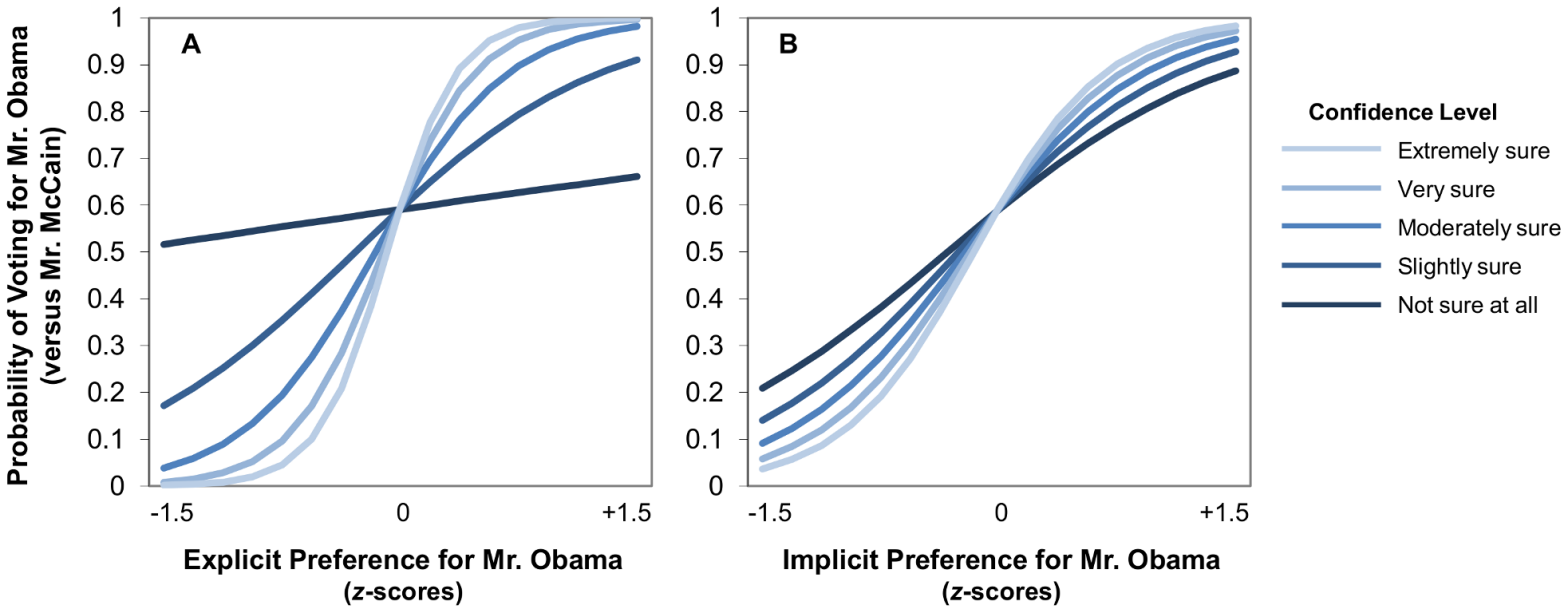
Simple slopes relating candidate attitudes to voting for respondents at each of the five levels of confidence. Probability of voting for Mr. Obama (1) versus Mr. McCain (0) as a function of candidate preference, confidence, and their interaction. Panel A: The association between explicit candidate preference and voting was moderated by confidence. Panel B: The association between implicit candidate preference and voting was not moderated by confidence.

Critically, the interaction between implicit candidate preference and confidence became non-significant (*B* = .362, *SE* = .239, *p* = .130). Implicit attitudes were predictive of voting behavior across the range of confidence. Further, the interaction between explicit candidate preference and confidence remained significant (*B* = 1.215, *SE* = .324, *p*<.001), indicating that explicit attitudes were more predictive of voting behavior at higher levels of confidence. To illustrate the nature of these relationships between attitudes and confidence, we calculated the simple slopes (displayed in Figure 1) relating implicit and explicit candidate preference to voting probabilities separately for respondents at each of the five levels of confidence. As confidence decreased, the predictive validity of explicit attitudes fell sharply, but the change for implicit attitudes was slight and non-significant.

To further examine the significant interaction between explicit attitudes and confidence, we tested the significance of those simple slopes (reported in Table 2). For voters at the four highest levels of confidence, explicit attitudes were a significant predictor of voting. However, at the lowest level of confidence (i.e., “not sure at all”), explicit attitudes were no longer significantly associated with voting. Though the simple slopes tests for implicit attitudes should be interpreted with caution given that the interaction between implicit attitudes and confidence was non-significant, it is clear that implicit attitudes were a significant predictor of voting across the full range of confidence ratings.

**Table 2:** Simple effects estimates for explicit and implicit candidate attitudes at each level of confidence.

	Explicit Candidate Attitudes				Implicit Candidate Attitudes			
Confidence Level	*B*	*p*	OR	95% CI	*B*	*p*	OR	95% CI
Extremely sure	4.590	<.001	98.477	27.965, 346.773	2.438	<.001	11.451	4.507, 29.095
Very sure	3.493	<.001	32.872	13.924, 77.606	2.111	<.001	8.259	4.368, 15.618
Moderately sure	2.395	<.001	10.971	5.228, 23.025	1.785	<.001	5.957	3.443, 10.307
Slightly sure	1.298	0.012	3.662	1.334, 10.052	1.458	<.001	4.297	2.042, 9.042
Not sure at all	0.201	0.788	1.222	0.282, 5.293	1.131	0.040	3.099	1.053, 9.120

Simple effects tests for predicted values of explicit and implicit candidate attitudes at each level of confidence. Corresponds to Model 3 in Table 1. *B*: regression weight *B* (log odds); OR: Odds ratio. Relative amount by which the odds increase (OR >1.0) or decrease (OR <1.0) when the value of the predictor is increased by 1 SD; 95% CI: 95% confidence interval for the odds ratio. Intervals that do not contain 1 are considered significant at *p*<.05.

Further, at the two lowest levels of confidence, the simple odds ratios for implicit attitudes were larger than those for explicit attitudes. To provide a more concrete illustration of these effect sizes, we will translate the predicted odds ratios into predicted probabilities. For those at the lowest level of confidence (i.e., “not sure at all” of their vote), the baseline probability of voting for Mr. Obama was 0.591, which corresponds to an odds ratio of 1.443. An increase of a single standard deviation in explicit preference for Mr. Obama uniquely increased those odds by a factor of 1.222 (i.e., 1.443×1.222 = 1.763), which translates to a probability of 0.638, while the same increase in implicit preference uniquely increased those odds by a factor of 3.099 (i.e., 1.443×3.099 = 4.472), which translates to a probability of .817. In short, “not sure at all” respondents who were one standard deviation above the mean on explicit preference for Mr. Obama had a predicted 64% chance of voting for him compared to the predicted 59% chance for those at the mean, while those who were one standard deviation above the mean on implicit preference had a predicted 82% chance of voting for Mr. Obama. Similarly, for those at the next lowest level of confidence (i.e., “slightly sure” of their vote), increases of a single standard deviation in explicit preference for Mr. Obama uniquely increased the chances of voting for him from 59% to 84%, while the same increase in implicit preference uniquely increased those chances to 86%. In other words, among more undecided voters, implicit candidate preference seemed to be a stronger predictor of voting behavior than explicit candidate preference.

However, it also should be noted that, at lower levels of confidence, despite the apparent differences in the magnitudes of the implicit and explicit attitudes estimates, the 95% confidence intervals for the pairs of estimates overlap substantially. For example, the 95% confidence interval for the simple odds ratio for the implicit candidate attitudes of “not sure at all” voters is 1.053 to 9.120, an interval which contains the simple odds ratio (1.222) for the explicit candidate attitudes of those same voters. Given the inappropriateness of calculating simple effects for implicit attitudes in the absence of a significant interaction with confidence, we are reluctant to draw firm conclusions regarding this finding. Nonetheless, this result suggests that, for voters who are more undecided, implicit and explicit attitudes are equally predictive of voting behavior.

#### Controlling for party affiliation and political ideology

In order to conduct a more conservative test, we repeated the Model 3 analysis with the inclusion of two explicit covariates: political party affiliation and political ideology. In Friese et al.'s [13] Study 2, when an additional explicit indicator of voting attitudes was included, all previously significant effects for implicit measures became nonsignificant. However, in our analysis, though both new covariates were significant predictors of voting behavior (*p*s<.001), the same pattern of results still emerged. Both explicit and implicit candidate preference were significant predictors of voting behavior (*p*s<.001), and explicit preference was moderated by confidence (*p* = .034), while implicit preference was not (*p* = .399). Additional information regarding these analyses is included in Table S1.

### Summary of Analysis 1

To summarize, as voters became less sure of their vote, explicit evaluations of the candidates became sharply less predictive of eventual voting. Implicit attitudes remained a significant predictor across the range of confidence, although the predictive effects of implicit attitudes decreased slightly. At high levels of confidence, explicit attitudes were a much stronger predictor of voting, but both explicit and implicit attitudes predicted voting independently. At low levels of confidence, implicit attitudes were slightly stronger predictors than explicit attitudes.

## Analysis 2

The same ANES data used for the candidate attitudes were also used for the examination of racial attitudes. In the analyses that follow, measures of voting behavior and confidence in the decision were identical to the analyses of candidate preferences described previously. Measures of confidence were selected from the wave that corresponded with the date of AMP administration and, as before, a control variable was included to indicate this factor.

### Measures

#### Implicit prejudice

Implicit attitudes toward Blacks were measured using the Affect Misattribution Procedure (AMP). Respondents completed the race AMP in either September or October 2008. Those who completed the candidates AMP in September completed the race AMP in October, and those who completed the candidates AMP in October completed the race AMP in September. The race AMP was administered according to the same display parameters used in the candidates AMP. Photographs of non-famous Black and White men, matched on attractiveness and perceived typicality of their racial group, were used as primes. Implicit prejudice was calculated by subtracting the proportion of pleasant judgments that followed photographs of Black individuals from the proportion of pleasant judgments that followed photographs of White individuals (*α* = .77). Higher scores indicate greater bias against Blacks (or in favor of Whites).

#### Explicit prejudice

We used all available explicit prejudice items that were administered before the election. The items included: (1) sympathy for Blacks, (2) admiration for Blacks, (3) perceptions that Blacks have too much political influence, (4) warm feelings toward Blacks, and (5) warm feelings toward Whites. The first three items were collected in September and the two feelings items were assessed in October. We subtracted the two feelings items to create a relative preference for Whites versus Blacks. All explicit items were then standardized and averaged into a composite, with higher scores reflecting more negative attitudes toward Blacks (*α* = .63).

### Results

This sample differs only slightly from the one used in the previous analysis. Of those respondents who completed all measures of interest (*N* = 2,024), 52.9% indicated a vote for Mr. Obama (47.1% for Mr. McCain). Additionally, 61.1% indicated that they were “extremely sure,” 18.3% that they were “very sure,” 13.0% that they were “moderately sure,” 4.1% that they were “slightly sure,” and 3.5% that they were “not sure at all.”

Explicit and implicit racial attitudes were modestly correlated (*r* = .282, *p*<.001), consistent with prior research [19], [21]. Table 3 presents the results of three binary logistic regression analyses predicting votes for Mr. Obama versus Mr. McCain from explicit and implicit racial attitudes and their interactions with confidence.

**Table 3:** Results of logistic regression analyses predicting voting behavior from explicit and implicit prejudice and confidence.

	Model 1: Explicit Prejudice					Model 2: Implicit Prejudice					Model 3: Explicit and Implicit Prejudice				
	% CCC = 66.7					% CCC = 56.3					% CCC = 67.2				
Variable	*B*	*SE*	Wald	*p*	OR	*B*	*SE*	Wald	*p*	OR	*B*	*SE*	Wald	*p*	OR
Constant	0.044	0.088	0.251	0.617	1.045	−0.011	0.085	0.015	0.901	0.989	0.025	0.091	0.075	0.784	1.025
Explicit Prejudice	−1.253	0.115	119.818	<.001	0.286						−1.212	0.120	102.620	<.001	0.298
Implicit Prejudice						−0.417	0.067	39.286	<.001	0.659	−0.250	0.077	10.543	0.001	0.779
Confidence	−0.071	0.069	1.073	0.300	0.931	−0.021	0.064	0.112	0.738	0.979	−0.073	0.069	1.103	0.294	0.930
Explicit*Confidence	−0.273	0.115	5.670	0.017	0.761						−0.257	0.117	4.795	0.029	0.774
Implicit*Confidence						−0.059	0.068	0.746	0.388	0.943	0.035	0.075	0.217	0.641	1.035

Predicting votes for Mr. Obama (1) versus Mr. McCain (0) from explicit and implicit prejudice toward Blacks and their interactions with confidence. Controlling for date of implicit attitude measure administration. Model 1 examines explicit prejudice separately (*N* = 2,056). Model 2 examines implicit prejudice separately (*N* = 2,024). Model 3 examines both prejudice measures simultaneously (*N* = 2,024). CCC: correctly classified cases; *B*: regression weight *B* (log odds); *SE*: standard error of the regression weight *B*; Wald: Wald test statistic; OR: Odds ratio. Relative amount by which the odds increase (OR >1.0) or decrease (OR <1.0) when the value of the predictor is increased by 1 SD.

When explicit attitudes were modeled separately (Model 1), respondents with more negative explicit attitudes toward Blacks were significantly less likely to cast a vote for Mr. Obama (*B* = −1.253, *SE* = .115, *p*<.001). Explicit attitudes also displayed a significant interaction with confidence (*B* = −.273, *SE* = .115, *p* = .017), indicating that explicit prejudice was more predictive of voting among decided voters than undecided voters.

When implicit attitudes were modeled separately (Model 2), individuals higher in implicit prejudice were less likely to vote for Mr. Obama (*B* = −.417, *SE* = .067, *p*<.001). However, there was no interaction between implicit attitudes and confidence (*B* = −.059, *SE* = .068, *p* = .388), indicating that implicit prejudice was predictive of voting among both decided and undecided voters. This finding replicates the results with implicit candidate attitudes when the variance due to explicit attitudes was removed. In the case of racial attitudes, implicit and explicit measures were only modestly correlated, making the shared variance less problematic. The next analysis examined the consequences of controlling for explicit attitudes.

**Figure 2:**
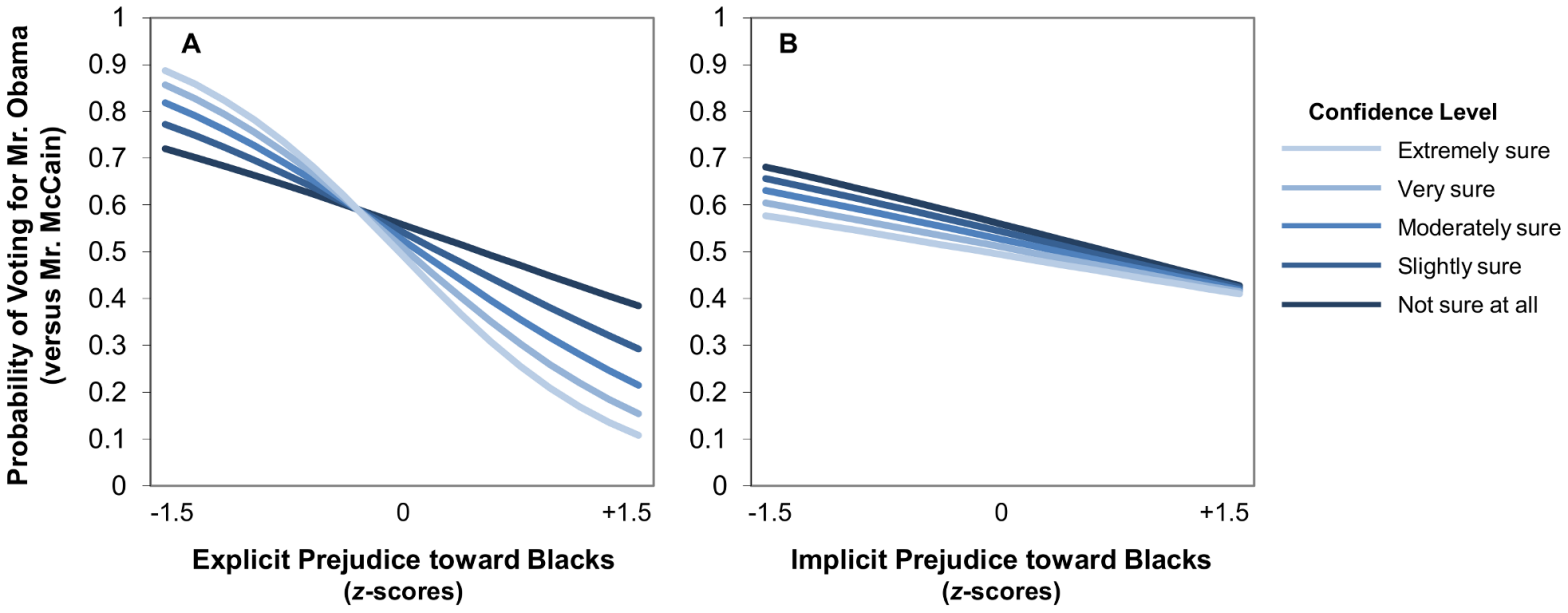
Simple slopes relating racial attitudes to voting for respondents at each of the five levels of confidence. Probability of voting for Mr. Obama (1) versus Mr. McCain (0) as a function of racial attitudes, confidence, and their interaction. Panel A: The association between explicit racial attitudes and voting was moderated by confidence. Panel B: The association between implicit racial attitudes and voting was not moderated by confidence.

When implicit and explicit attitudes were modeled simultaneously (Model 3), both explicit attitudes (*B* = −1.212, *SE* = .120, *p*<.001) and implicit attitudes (*B* = −.250, *SE* = .077, *p* = .001) uniquely predicted voting. The interaction between implicit attitudes and confidence remained non-significant (*B* = .035, *SE* = .075, *p* = .641), and the interaction between explicit attitudes and confidence remained significant (*B* = −.257, *SE* = .117, *p* = .029). To illustrate the nature of these relationships between prejudice and confidence, we plotted the simple slopes relating implicit and explicit racial attitudes to voting probabilities separately for respondents at each level of confidence (displayed in Figure 2). Explicit prejudice had a larger overall predictive effect than implicit attitudes. However, only explicit attitudes were moderated by confidence.

Additionally, to examine further the significant interaction between explicit prejudice and confidence, we tested the significance of those simple slopes (Table 4). For voters at the four highest levels of confidence, explicit prejudice was a significant predictor of voting. However, at the lowest level of confidence, explicit prejudice was not significantly associated with voting. While the simple effects for implicit prejudice remain weaker than those for explicit prejudice at each level of confidence, they vary only slightly across the range of confidence (simple odds ratios from .705 to .798), suggesting that implicit attitudes remain a consistent predictor regardless of confidence level. (Simple slopes for implicit attitudes should be interpreted with caution because there was no significant interaction.) At lower levels of confidence, the simple odds ratios for both explicit and implicit attitudes are comparable (e.g., .624 for explicit versus .705 for implicit for those who were “not sure at all”).

**Table 4:** Simple effects estimates for explicit and implicit racial attitudes at each level of confidence.

	Explicit Racial Attitudes				Implicit Racial Attitudes			
Confidence Level	*B*	*p*	OR	95% CI	*B*	*p*	OR	95% CI
Extremely sure	−1.392	<.001	0.248	0.186, 0.332	−0.225	0.015	0.798	0.666, 0.958
Very sure	−1.162	<.001	0.313	0.247, 0.397	−0.256	0.001	0.774	0.664, 0.902
Moderately sure	−0.932	<.001	0.394	0.281, 0.552	−0.287	0.010	0.750	0.602, 0.935
Slightly sure	−0.702	0.007	0.496	0.299, 0.822	−0.319	0.057	0.727	0.524, 1.009
Not sure at all	−0.472	0.184	0.624	0.311, 1.251	−0.350	0.126	0.705	0.450, 1.104

Simple effects tests for predicted values of explicit and implicit racial attitudes at each level of confidence. Corresponds to Model 3 in Table 3. *B*: regression weight *B* (log odds); OR: Odds ratio. Relative amount by which the odds increase (OR >1.0) or decrease (OR <1.0) when the value of the predictor is increased by 1 SD; 95% CI: 95% confidence interval for the odds ratio. Intervals that do not contain 1 are considered significant at *p*<.05.

As we did before in Analysis 1, we can provide a more concrete illustration of the effect sizes by translating the predicted odds ratios into predicted probabilities. For “not sure at all” voters in Model 3, a single standard deviation increase in explicit prejudice uniquely decreased the probability of voting for Mr. Obama from 56% to 44%, while a single standard deviation increase in implicit prejudice decreased that probability to 47%. Similarly, for “slightly sure” voters, increases in explicit prejudice uniquely decreased the probability of voting for Mr. Obama from 54% to 37%, while increases in implicit prejudice decreased that probability to 46%. In other words, among more undecided voters, explicit prejudice seemed to be a stronger predictor of voting behavior than implicit prejudice. However, it also should be noted that, at the lowest level of confidence, despite the apparent difference in the magnitudes of the implicit and explicit attitudes estimates, the 95% confidence intervals for the pair of estimates overlap substantially, suggesting that, for voters who are more undecided, implicit and explicit attitudes are equally predictive of voting behavior.

### Summary of Analysis 2

Together, the results of the racial attitudes analyses are generally consistent with attitudes toward the candidates: Explicit attitudes were more predictive of voting for decided than undecided voters, but implicit attitudes were similarly predictive for undecided and decided voters.

## Analysis 3

One reason that implicit attitudes should predict behavior among the undecided is that when people introspect about whether they have reached a decision, they attend primarily to consciously endorsed attitudes and neglect implicit attitudes. This does not imply that implicit attitudes are necessarily unconscious, but simply that people tend to consider them to be a less valid basis for judgments [5], [7]. To test the hypothesis more directly, we next examined the relationships between attitude extremity and confidence. We expected that more extreme explicit attitudes would be associated with greater confidence in one's vote, but that this association would be weaker for implicit attitudes. Statistically, this hypothesis predicts a curvilinear relationship between explicit attitudes and confidence.

### Candidate Attitudes

**Figure 3:**
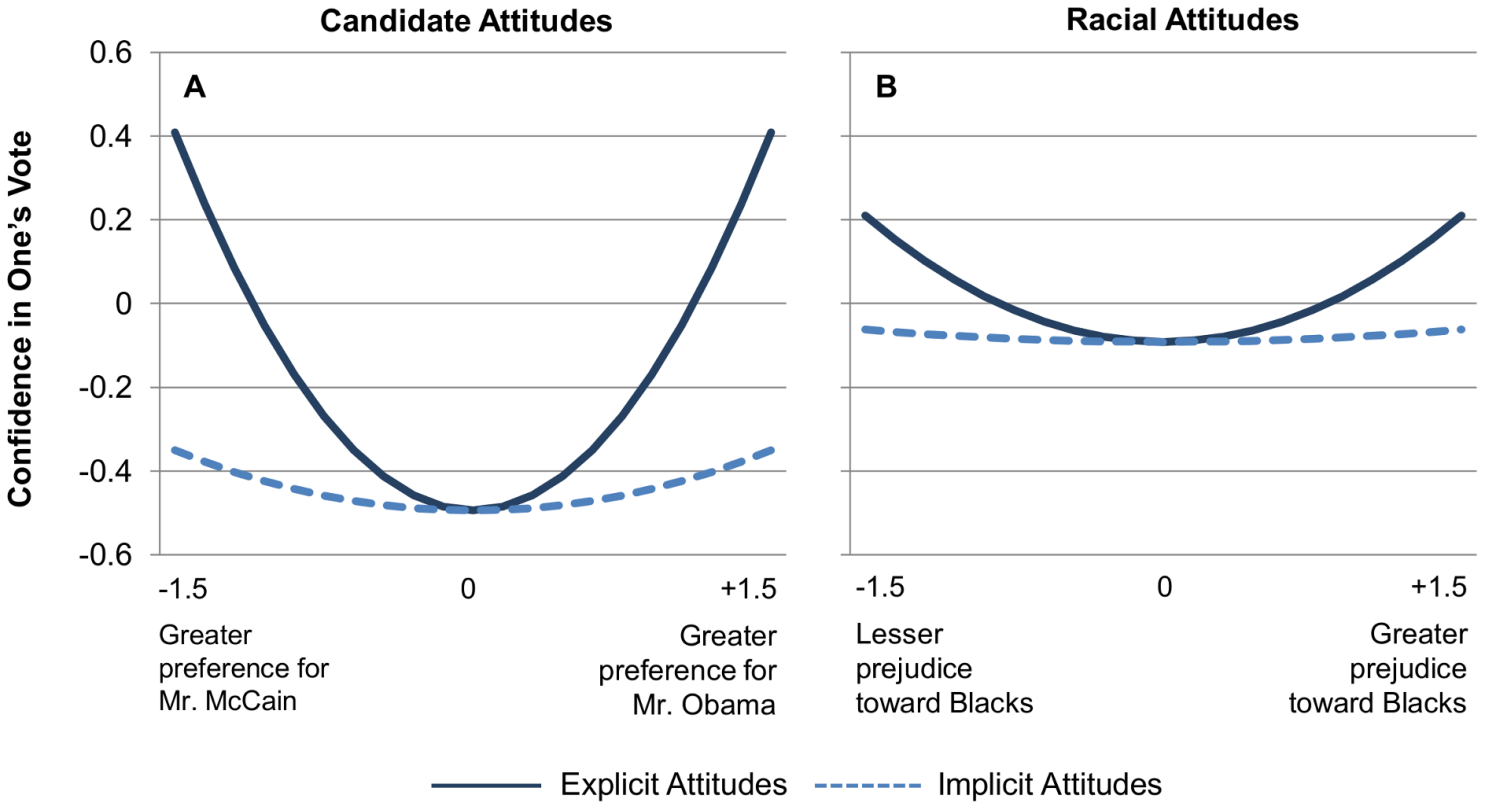
Quadratic relationships between confidence in one's vote and explicit and implicit attitudes. Panel A: The quadratic relationship between confidence and explicit candidate attitudes was larger than the quadratic relationship between confidence and implicit candidate attitudes. Panel B: The quadratic relationship between confidence and explicit prejudice was larger than the quadratic relationship between confidence and implicit prejudice.

To test this hypothesis, we regressed confidence on explicit and implicit candidate preferences and their quadratic terms (see Figure 3, Panel A). While the linear effects for explicit attitudes (*B* = .023, *SE* = .029, *p* = .428) and implicit attitudes (*B* = .001, *SE* = .032, *p* = .985) were non-significant, both quadratic effects were significant. More extreme explicit attitudes in favor of either candidate were strongly associated with greater confidence (*B* = .401, *SE* = .021, *p*<.001). A similar but much weaker relationship was observed for implicit candidate attitudes (*B* = .064, *SE* = .017, *p*<.001). Importantly, the 95% confidence interval for explicit attitudes (.359 to .443) did not include the coefficient for implicit attitudes, indicating that the quadratic term for explicit attitudes was significantly greater than that for implicit attitudes.

### Racial Attitudes

We conducted a parallel analysis for racial attitudes (see Figure 3, Panel B). The linear effect of explicit prejudice was significant, indicating that higher explicit prejudice was slightly associated with lower confidence (*B* = −.142, *SE* = .036, *p*<.001). More importantly, the quadratic effect of explicit attitudes was also significant, indicating that more extreme racial attitudes (either pro-Black or anti-Black) were associated with greater confidence in one's voting intention (*B* = .134, *SE* = .028, *p*<.001). For implicit race attitudes, neither the linear effect (*B* = −.021, *SE* = .027, *p* = .437) nor the quadratic effect (*B* = .013, *SE* = .011, *p* = .227) were significant.

### Summary of Analysis 3

These analyses are consistent with the hypothesis that, when people introspect about whether they have decided, they focus on explicit attitudes. As a result, more extreme explicit attitudes are associated with greater confidence. Implicit attitudes, in contrast, may be overlooked when people assess whether they have reached a confident decision, especially if they are considered a less trusted or valid source of information. This asymmetry between explicit and implicit attitudes provides new evidence for a potential mechanism that may help explain why implicit attitudes predict future behavior among undecided voters. If strong implicit attitudes have significant effects on behavior but small effects on assessments of confidence, then implicit attitudes should predict behavior even among undecided voters.

## General Discussion

The idea that implicit attitude measures can predict the behavior of undecided voters has generated a great deal of interest, because it suggests that precursors of decisions can be detected before respondents feel they have made up their minds. This idea has also generated some disagreement over whether and when such effects might be expected, as some prior studies have found implicit attitudes to predict voting among undecided voters whereas others have not. We re-examined these ideas using a large nationally representative sample and a longitudinal design, with implicit measures of both candidate attitudes and racial attitudes. We found that when implicit and explicit attitudes were highly correlated, as in the case of attitudes toward political candidates, the role of implicit attitudes depended critically on whether explicit attitudes were statistically controlled. When explicit candidate attitudes were not controlled, implicit candidate attitudes were more predictive among decided than undecided voters. However, this finding appears to result from the shared variance between implicit and explicit candidate attitudes. When explicit candidate attitudes were controlled, implicit candidate attitudes were equally predictive for decided and undecided voters. The results for candidate attitudes and racial attitudes both provided evidence that implicit attitudes may predict the behaviors of voters who feel that they have not made up their minds.

We also explored the hypothesis that implicit attitudes might predict behavior even among the undecided because, when people introspect, they attend to consciously endorsed attitudes and neglect implicit attitudes. Consistent with this idea, we found that more extreme explicit attitudes were associated with greater confidence. This association suggests an explanation for why explicit attitudes lost so much of their predictive power at lower levels of confidence: Weaker explicit attitudes are both less predictive of behavior and more likely to generate metacognitive judgments of low confidence. The relationship between attitudes extremity and confidence was much weaker for implicit attitudes. We also found that this effect was much larger for candidate attitudes than racial attitudes. This difference may result because, when people assess their confidence in their vote, they are most likely to introspect about their feelings toward the candidates. However, the fact that a similar (albeit weaker) tendency was found for explicit prejudice suggests that people may also have consulted their explicit feelings toward Blacks in general.

This pattern may also be consistent with Galdi and colleagues' [2], [4] biased processing hypothesis. By that account, confidence accrues to the extent that confirmatory evidence is available to support one's biased processing. Thus, the same biased processing that leads to deliberate decisions may also lead to higher confidence. If we had failed to find evidence that confidence was more strongly associated with explicit than implicit attitudes, it would have cast doubt on the introspective neglect hypothesis. These curvilinear relationships offer positive support for the introspective neglect hypothesis, but they do not distinguish between that account and the biased processing account. Further, though these results are suggestive, they do not provide direct evidence for the causal mechanism presumed to underlie the introspective neglect hypothesis, namely that implicit attitudes do not factor into metacognitive judgments of decidedness because they are considered a less valid source of information. Future research should directly test this causal hypothesis.

### Theoretical Significance

The present results shed light on an important asymmetry between how explicit and implicit attitudes predict behavior. Some perspectives suggest that implicit attitudes may only predict behaviors that are spontaneous or difficult to control, whereas explicit attitudes predict deliberate behavior [22]. Other authors have pointed out that a number of different patterns have been documented in the literature, including additive effects, indirect effects, and interactive effects [23]. The present results are consistent with both indirect effects and additive direct effects. Indirect effects of implicit attitudes through explicit attitudes are suggested by the fact that controlling for explicit attitudes reduced the predictive effects of implicit attitudes. And yet, after controlling for explicit attitudes, we found consistent unique effects of implicit attitudes.

Both indirect and direct pathways are important for understanding the causal forces that shape voting and other political behavior. Indirect pathways suggest that implicit attitudes influence the thoughts and feelings that will eventually be explicitly endorsed, through mechanisms such as biased processing of confirmatory evidence [3]. Even when explicit attitudes are the proximal cause, they may be the result of earlier implicit processes. Indirect effects are potentially important evidence for the purpose of understanding such causal chains. Direct effects are also important, but they suggest different processes. For example, prominent theoretical models suggest that highly accessible attitudes may influence decisions by influencing how the options are construed even if respondents are motivated to deny those attitudes explicitly [24]. Such effects would appear in the present data as a direct effect of implicit attitudes. Both direct and indirect effects provide evidence toward understanding the multiple pathways by which implicit and explicit attitudes may influence consequential behavior.

### Practical Significance

Do implicit measures add substantively to the ability to forecast election outcomes? Explicit attitude measures are simpler and easier to administer than implicit measures, so implicit measures would have to show a non-trivial effect beyond explicit measures to justify including them for some practical purposes. If we were simply interested in whether implicit measures improve election forecasts for *all* voters, then overall measures of goodness of fit can provide us with the necessary information. For example, Table 1 shows one such measure of goodness of fit, the percentage of correctly classified cases, for the analyses involving candidate attitudes, while Table 3 shows the same information for the analyses involving racial attitudes. In both sets of analyses, though implicit attitudes are significant unique predictors of voting behavior, the models including both explicit and implicit attitudes (Models 3) do not improve substantially on the models including just explicit attitudes (Models 1). The increase in the percentage of correctly classified cases is 2.4% for candidate attitudes and just 0.5% for racial attitudes.

However, we are particularly interested in whether implicit measures improve election forecasts for a *subset* of voters, those who are less sure of their vote, to which these overall measures of goodness of fit cannot speak. To better address this specific question, we turn to a comparison of the model-implied simple effects estimates for both explicit and implicit attitudes at lower levels of confidence. (See Tables 2 and 4.) For candidate attitudes, the greater magnitude and significance of the simple effects estimates for implicit attitudes compared to explicit attitudes suggests that implicit attitudes may be more predictive than explicit attitudes among voters who are more undecided. However, the reverse is true of racial attitudes; in this case, explicit attitudes may be more predictive than implicit attitudes among voters who are more undecided. Further, in both sets of analyses, the confidence intervals constructed around the simple odds ratios lead to the conclusion that, at lower levels of confidence, implicit and explicit attitudes are equally predictive of voting behavior.

Overall, explicit attitudes were clearly substantial predictors of the vote. Taking all these results together, we believe that the data suggest that implicit and explicit attitudes are *equally* predictive for less confident voters. Nonetheless, these conclusions are drawn from models in which implicit measures showed substantial and unique predictive power after controlling for explicit attitudes. This is especially so among undecided voters, for whom explicit measures lose some of their predictive power. Therefore, including implicit measures in studies of voting behavior seems potentially useful to election forecasters.

### Relationship to Past Research

A close look at the results of Friese et al. [13] shows that, in the three analyses in which they entered implicit and explicit attitudes simultaneously (reported in their Tables 3, 5, and 7), the interaction between implicit attitudes and decidedness was not significant, consistent with the present results. Their interpretations differed from ours, however, because their study found that, once explicit measures were included, implicit attitudes were not consistently a significant predictor of voting. That is, in some analyses they found that implicit attitudes predicted voting for neither decided nor undecided voters after explicit attitudes were controlled, whereas in other analyses they found significant but small effects of implicit attitudes for both decided and undecided voters. Overall, the qualitative pattern of results in the Friese et al. study and ours is quite similar. The main difference is that we found a consistent unique effect of implicit attitudes whereas they found a less consistent effect.

Several procedural differences could potentially explain the discrepancies between these studies. Both studies used large samples, but only our sample was representative of the population. The two studies used different implicit measures and different measures of decidedness. Given these procedural differences, the parallels between the conclusions are rather more striking than their divergences. These explanations are necessarily speculative, and future research should systematically compare procedures to clarify the conditions under which implicit attitudes are most and least predictive.

Our findings are consistent with the general proposal by Galdi and colleagues [2] that implicit attitudes may be valuable for predicting the behavior of undecided voters. Our findings were not entirely consistent, however, with the particulars of Galdi et al.'s hypothesis or with the mechanism that they proposed. Firstly, the two predictions that can be derived from Galdi et al.'s original work are (1) that implicit attitudes are more predictive of voting behavior than explicit attitudes for undecided voters; and (2) that implicit attitudes are more predictive of voting behavior for undecided than decided voters. On the first point, our analyses do not firmly support that conclusion. In fact, the body of evidence suggests only that implicit and explicit attitudes are equally predictive of voting behavior for voters who are more undecided. Further, our findings do not support the second point: In our analyses, the nonsignificant interactions between implicit attitudes and confidence indicate instead that implicit attitudes are equally predictive of voting behavior for voters who are more or less decided.

Further, Galdi and colleagues' [2], [4] biased processing account implies that implicit attitudes only affect deliberate decisions indirectly, through the biased processing of subsequent information. By the time a person has gathered enough confirmatory evidence to feel confident in their decision, there should be no direct effect of implicit attitudes after controlling for the effects of explicit attitudes. This account thus predicts no unique effect of implicit attitudes among decided voters. However, we found that implicit attitudes were predictive among both decided and undecided voters and that, if anything, their effects were slightly stronger among decided voters. This pattern is consistent with the hypothesis that people neglect implicit attitudes and focus on explicitly endorsed attitudes when they introspect about their confidence in decisions. Our findings do not rule out the hypothesis that biased processing contributes to the downstream effects of implicit attitudes. Both biased processing and introspective asymmetries may play a role in explaining why implicit attitudes interact with confidence differently than explicit attitudes.

Earlier research analyzed the racial attitudes data from the ANES and found that explicit and implicit racial attitudes each uniquely predicted voting, although the effects of explicit prejudice were larger [25], [26]. Two recent papers re-examined the role of implicit prejudice in the ANES data and concluded that, whereas the effects of explicit prejudice were large and important, the unique influence of implicit prejudice was not large enough to be politically consequential [27], [28]. None of these previous studies, however, considered whether voters were decided.

The present study suggests an interesting new perspective on the relative impact of implicit and explicit attitudes. By the time implicit attitudes were measured in the ANES (September–October), most respondents had reached a confident decision. The analyses in the present article suggest that the relative importance of explicit and implicit attitudes will depend on the proportion of the population who has reached a firm decision. Though one conclusion may be that explicit prejudice is generally more consequential for voting, an alternative hypothesis is that, when undecided voters are included in greater numbers, the relative effect size of implicit attitudes should increase. Future research should test this hypothesis by measuring implicit and explicit attitudes earlier in elections when fewer respondents have reached decisions.

## Conclusion

Implicit attitudes have been suggested as a key to unlock the hidden preferences of undecided voters. Past research, however, offered mixed support for this hypothesis. The present research found that, when the influence of explicit attitudes was controlled, implicit attitudes predicted voting as well for the undecided as for the decided voters. Implicit tests may offer a useful tool in forecasting elections, especially at early stages when many voters have yet to make up their minds.

## Supporting Information

Table S1 **Results of logistic regression analysis predicting voting behavior from explicit and implicit candidate preference and confidence, while including two additional explicit indicators of voting attitudes.** Predicting votes for Mr. Obama (1) versus Mr. McCain (0) from explicit and implicit preference for Mr. Obama (versus Mr. McCain) and their interaction with confidence. Controlling for date of attitude measures administration, political ideology, and party affiliation. Corresponds to Table 1, Model 3 (see main manuscript for details). Political ideology and party affiliation were both assessed in October 2008 using 6-point scales ranging from *extremely liberal* to *extremely conservative* and *strong Democrat* to *strong Republican*, respectively. All continuous variables have been standardized using *z*-scores. *N* = 1,977. Correctly classified cases = 93.2%. *B*: regression weight *B* (log odds); *SE*: standard error of the regression weight *B*; Wald: Wald test statistic; OR: Odds ratio. Relative amount by which the odds increase (OR >1.0) or decrease (OR <1.0) when the value of the predictor is increased by 1 SD.
(DOCX)
