# Correction: Decisions among the Undecided: Implicit Attitudes Predict Future Voting Behavior of Undecided Voters

**DOI:** 10.1371/journal.pone.0105655

**Published:** 2014-08-11

**Authors:** The PLOS ONE Staff

The following information is missing from the Funding section: Research funding was provided by Russell Sage Foundation Grant #83-13-06. The funder had no role in study design, data collection and analysis, decision to publish, or preparation of the manuscript.
