# Supplementary material for: Decisions among the Undecided: Implicit Attitudes Predict Future Voting Behavior of Undecided Voters
Source: PLoS One. 2014 Jan 29;9(1):e85680. doi: 10.1371/journal.pone.0085680 (PMC3906000; doi:10.1371/journal.pone.0085680)
Supplement: Table S1 — Results of logistic regression analysis predicting voting behavior from explicit and implicit candidate preference and confidence, while including two additional explicit indicators of voting attitudes. Predicting votes for Mr. Obama (1) versus Mr. McCain (0) from explicit and implicit preference for Mr. Obama (versus Mr. McCain) and their interaction with confidence. Controlling for date of attitude measures administration, political ideology, and party affiliation. Corresponds to Table 1, Model 3 (see main manuscript for details). Political ideology and party affiliation were both assessed in October 2008 using 6-point scales ranging from extremely liberal to extremely conservative and strong Democrat to strong Republican, respectively. All continuous variables have been standardized using z-scores. N = 1,977. Correctly classified cases = 93.2%. B: regression weight B (log odds); SE: standard error of the regression weight B; Wald: Wald test statistic; OR: Odds ratio. Relative amount by which the odds increase (OR >1.0) or decrease (OR <1.0) when the value of the predictor is increased by 1 SD. (DOCX) [file pone.0085680.s001.docx]

**Table S1.**

| **Variable** | ***B*** | ***SE*** | **Wald** | ***p*** | **OR** |
| --- | --- | --- | --- | --- | --- |
| Constant | 0.398 | 0.268 | 2.208 | 0.137 | 1.489 |
| Explicit Attitudes | 2.981 | 0.458 | 42.349 | <.001 | 19.707 |
| Implicit Attitudes | 1.696 | 0.371 | 20.956 | <.001 | 5.452 |
| Confidence | -0.031 | 0.122 | 0.062 | 0.803 | 0.970 |
| Explicit*Confidence | 0.810 | 0.374 | 4.686 | 0.030 | 2.248 |
| Implicit*Confidence | 0.221 | 0.262 | 0.713 | 0.399 | 1.248 |
| Political Ideology | -0.746 | 0.209 | 12.789 | <.001 | 0.474 |
| Party Affiliation | -1.243 | 0.206 | 36.433 | <.001 | 0.289 |
